# Adult Midgut Malrotation With Chronic Volvulus With Superior Mesenteric Artery (SMA) Thrombosis: A Recherche

**DOI:** 10.7759/cureus.43754

**Published:** 2023-08-19

**Authors:** Dhivakar S, Sudhir K Singh, Asish Das, Sanjay Katragadda, Ashish Mishra

**Affiliations:** 1 General Surgery, All India Institute of Medical Sciences, Rishikesh, IND

**Keywords:** thrombosis, superior, mesenteric artery, intestinal volvulus, intestinal malrotation

## Abstract

Intestinal malrotation is primarily a surgical condition of neonates due to abnormal intestinal rotation during fetal development. Usually, the presentation is immediately after birth. Adult midgut malrotation is rare and primarily detected at laparotomy or incidental radiological imaging for various conditions. We report a sporadic case of a 35-year-old male who presented to the surgical outpatient department (OPD) complaining of dull aching abdominal pain after taking meals for two months. He was able to tolerate a liquid diet only and able to carry out his routine work comfortably. In imaging studies, it was found to be a case of midgut malrotation with volvulus and superior mesenteric artery (SMA) thrombosis with collaterals without features of intestinal obstruction. The patient underwent diagnostic laparoscopy, and a midgut volvulus was identified with Ladd’s bands. He underwent exploratory laparotomy with Ladd’s procedure. Postoperatively symptoms were resolved, and the patient was discharged in stable condition.

If intestinal malrotation presents in adults, it is challenging to diagnose it as it presents with atypical symptoms like chronic vague abdominal pain and weight loss. Often radiological correlation is essential to diagnose such patients. For surgical intervention, a laparoscopic approach is considered better in expert hands. Even though the disease has a chronic course, a high index of suspicion should arise when treating such cases of intestinal malrotation in an adult male. Timely surgery can do miracles and prevent catastrophic complications.

## Introduction

The incidence of intestinal malrotation is estimated to be 1 in 6000 live births [1]. Neonates present with features of bilious vomiting as early as the first week, and 60%-85% of them are diagnosed in the first month of life. Most (90%) patients present within one year of life [1-19]. Midgut malrotation happens due to anomalous partial or complete non-rotation when the midgut herniates into the proximal umbilical cord around the 10-12th week of gestation and fails to rotate 270⁰ counter-clockwise against the axis of the superior mesenteric artery (SMA) [2]. Presentation in adulthood is a rarity and is reported to be around 0.2-0.5%, of which 15% are present as midgut volvulus [3]. Most patients are asymptomatic; hence, the true incidence of midgut malrotation is not fully understood. A literature review accounted for only 92 symptomatic cases so far [4].

Symptoms, if present, are often due to peritoneal bands between the cecum and right lateral abdominal wall, namely earlier by Ladd in 1932 as Ladd’s bands. Patients can present with acute abdominal symptoms suggesting bowel ischemia or obstruction due to a midgut or cecal volvulus. Some patients also present with vague, chronic abdominal complaints. Thus diagnosing intestinal malrotation in adults is highly complicated. According to existing literature, approximately 100 patients have been reported to present as midgut volvulus, often diagnosed with contrast-enhanced computed tomography (CECT) showing hallmark features like a whirlpool sign, corkscrewing of the proximal gut, and abnormally oriented SMA and superior mesenteric vein (SMV) [4]. The treatment of choice always includes a surgical procedure using Ladd operation, even in asymptomatic individuals. The role of doing a concurrent appendectomy is still controversial. However, an appendectomy can be considered to avoid future diagnostic confusion due to appendicitis [5].

Our patient was a case of intestinal malrotation with chronic intermittent midgut volvulus and SMA thrombosis who was asymptomatic for an extended period and presented with vague abdominal symptoms. Navarini et al. have published a case study of a 54-year-old female who presented with chronic abdominal pain and was diagnosed with intestinal malrotation with SMA and SMV thrombosis. To the best of our search, only a handful of cases have been published where adult patients of malrotation had SMA or SMV thrombosis concurrent with volvulus making it a rare finding [6-7].

## Case presentation

A 35-year-old male, a daily wage laborer, presented to the surgical outpatient department (OPD) with complaints of abdominal pain for two months. The pain was dull and aching, aggravated after food intake, which was relieved with over-the-counter medications but reappeared with food intake. The pain was associated with intermittent non-bilious vomiting, usually a few hours after food intake. His appetite was preserved, but he had a significant weight loss of 12 kg for the past two months. There was no history of prior hospital admissions for similar complaints. He had no addictions to smoking, alcohol, or any drug. He was not having any comorbidities or a history of abdominal surgeries in the past. There was no history suggestive of any intestinal obstruction in the past. On general physical examination, the patient was conscious and oriented to time, place, and person. His vital were: pulse rate of 80 bpm, blood pressure of 120/90 mmHg, and respiratory rate of 20 per minute. He was thin and built with a body mass index (BMI) of 19. Abdominal examination revealed a non-tender scaphoid abdomen and no organomegaly. Other systemic examinations were within normal limits.

**Figure 1:**
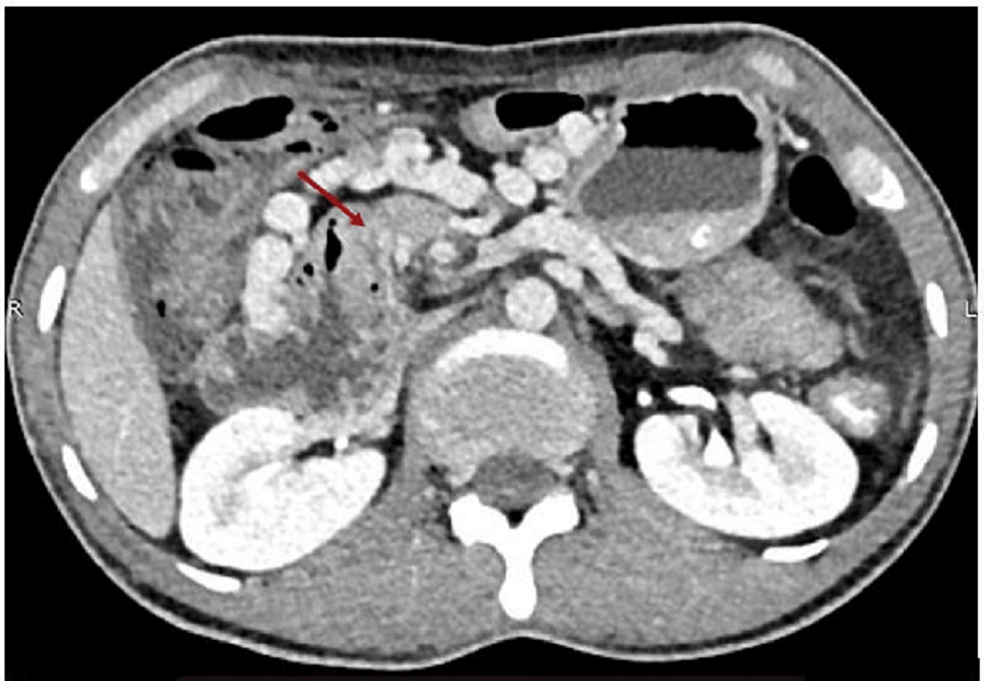
Third part of the duodenum rotating around SMA (red arrow). SMA, superior mesenteric artery

**Figure 2:**
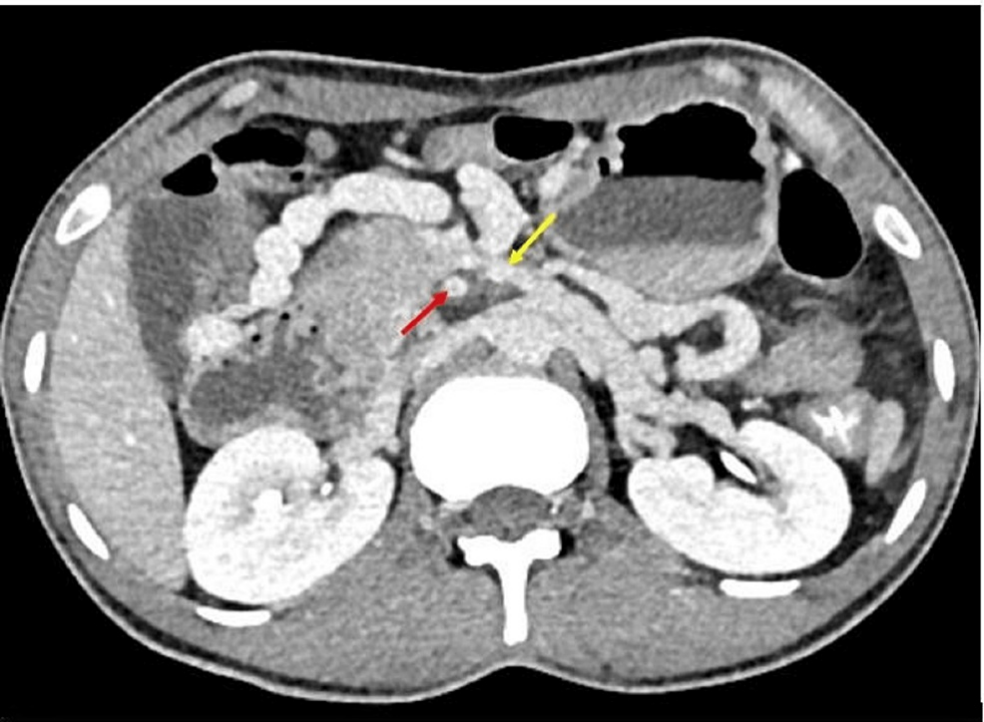
SMV (yellow arrow) left to the SMA (red area). SMV, superior mesenteric vein; SMA, superior mesenteric artery

**Figure 3:**
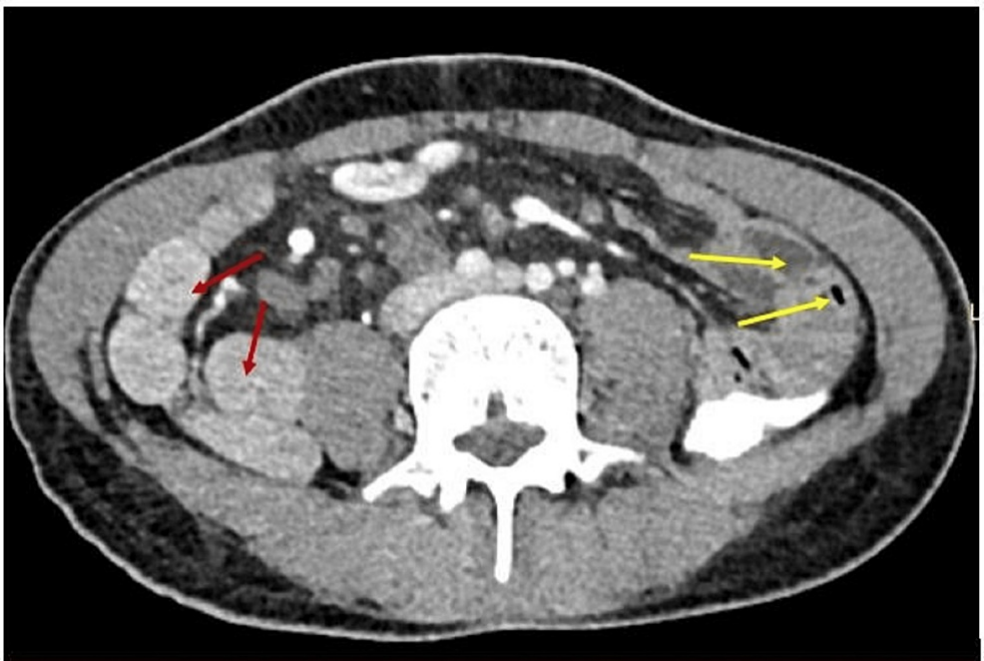
The small bowel loops (red arrow) mostly on the right side and large bowel loops (yellow arrow) on the left side.

**Figure 4:**
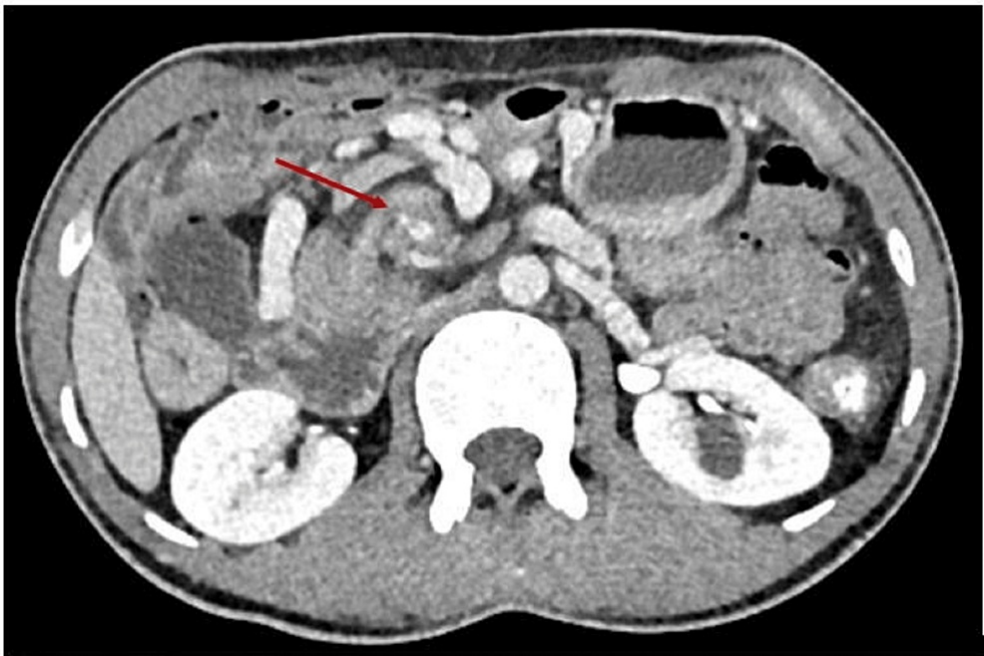
Whirlpool sign (red arrow).

**Figure 5:**
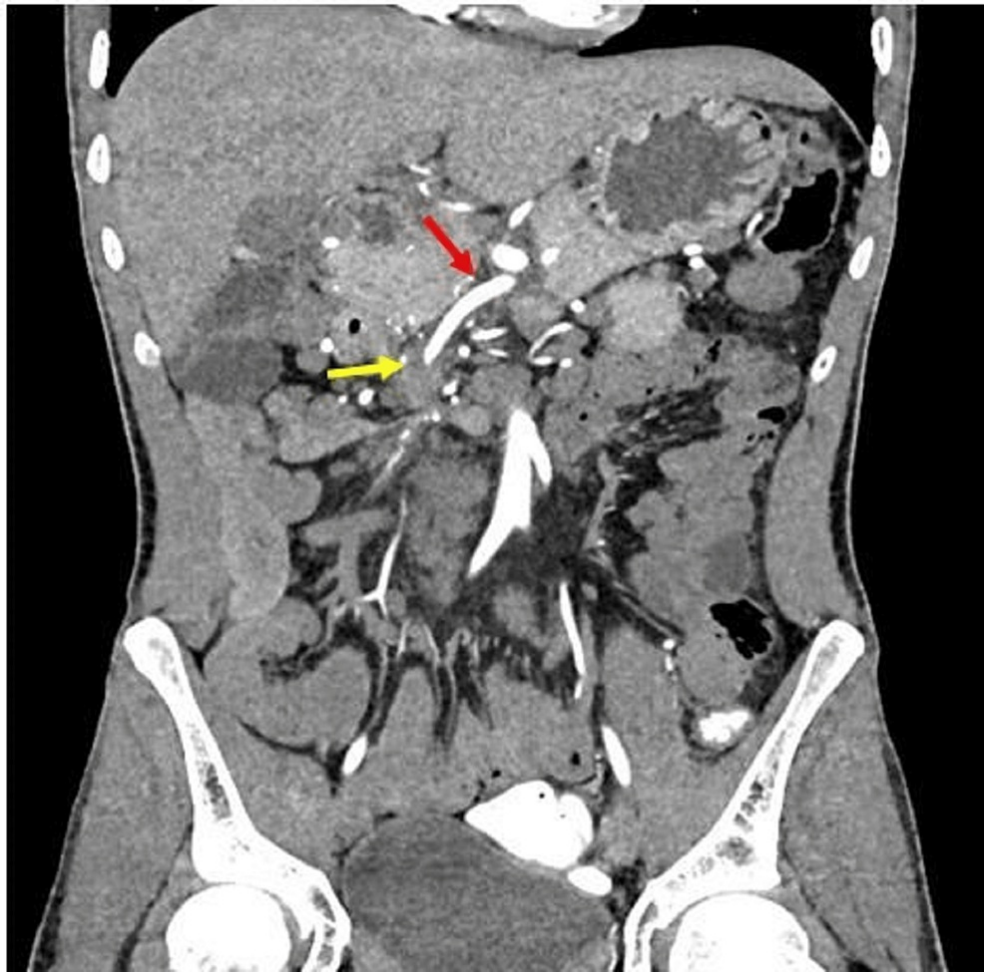
SMA is opacifying (red arrow) with no opacification at the area of volvulus (yellow arrow) and getting opacified by collaterals distal to the thrombus. SMA, superior mesenteric artery

Routine blood investigations were within normal limits. Contrast-enhanced computed tomography (CECT) abdomen revealed a duodenojejunal junction lying to the right side of the spine with clockwise twisting of bowel loops, SMA, superior mesenteric vein (SMV), and mesentery. The relationship between SMA and SMV was inverted. There was gradual attenuation of SMA with complete occlusion at the level of the twisted segment. The SMV was not visualized beyond the twisted segment, with multiple dilated collaterals forming from the portal vein and proximal SMV in the subhepatic and umbilical region. The ileocecal junction was pulled up and lying to the right of the midline at the L2-L3 vertebrae level. The twisted midgut bowel loops showed normal contrast opacification with no signs of bowel ischemia (Figures 1-5).

**Figure 6:**
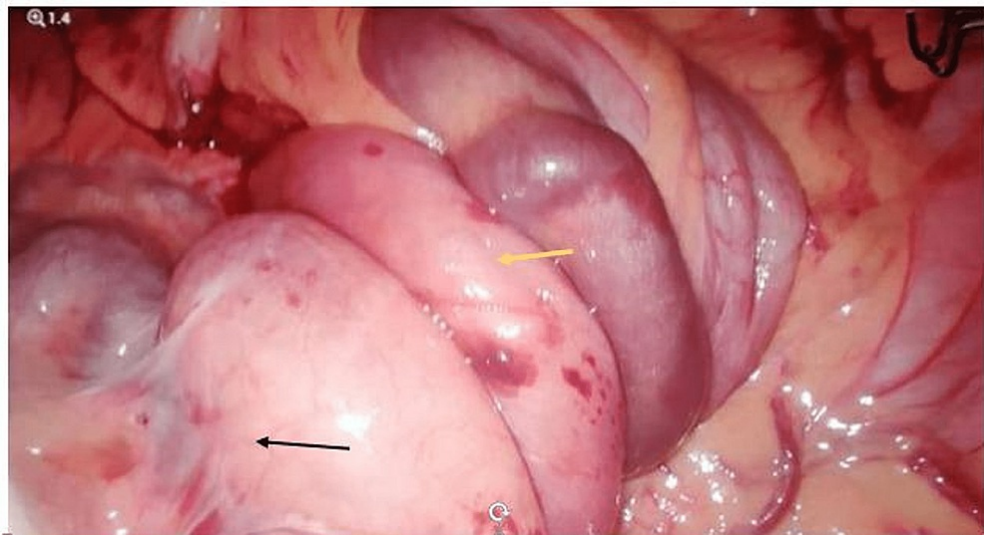
Laparoscopic image showing rotated small bowel loops (yellow arrow) on its mesentery and band (black arrow).

The patient underwent diagnostic laparoscopy, which confirmed the findings of the CT abdomen. There was subhepatic cecum with midgut malrotation and volvulus with multiple tortuous dilated collaterals (Figure 6).

**Figure 7:**
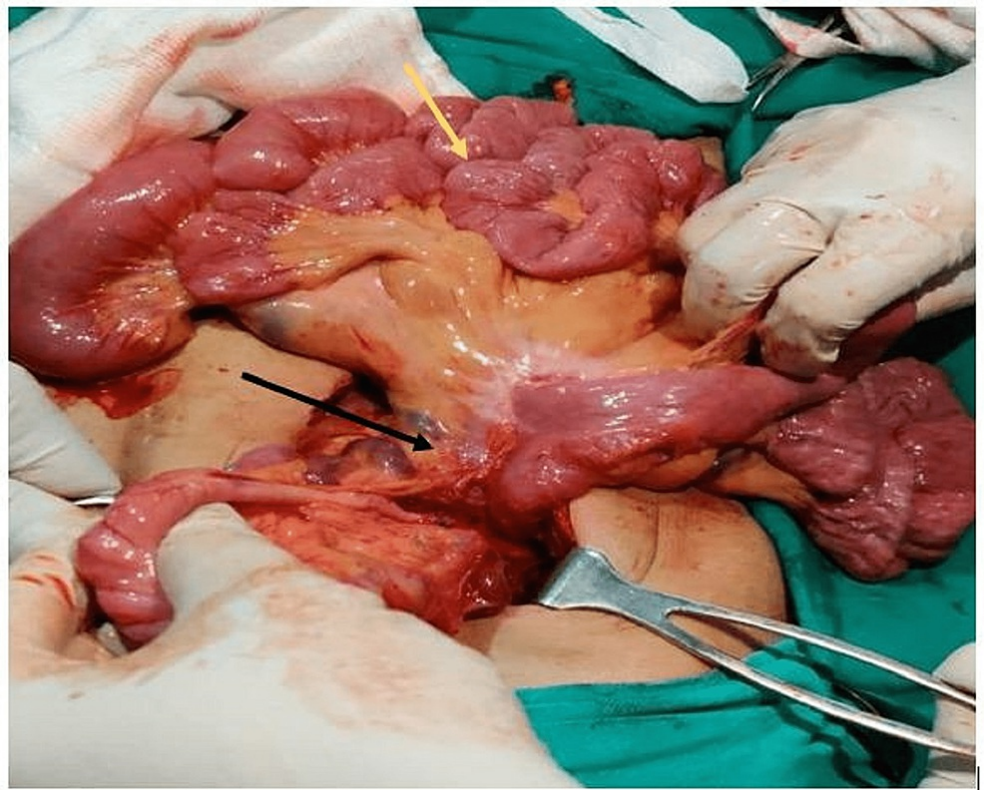
After derotation of the mesentery (black and yellow arrow showing root of mesentery and small bowel loops, respectively).

**Figure 8:**
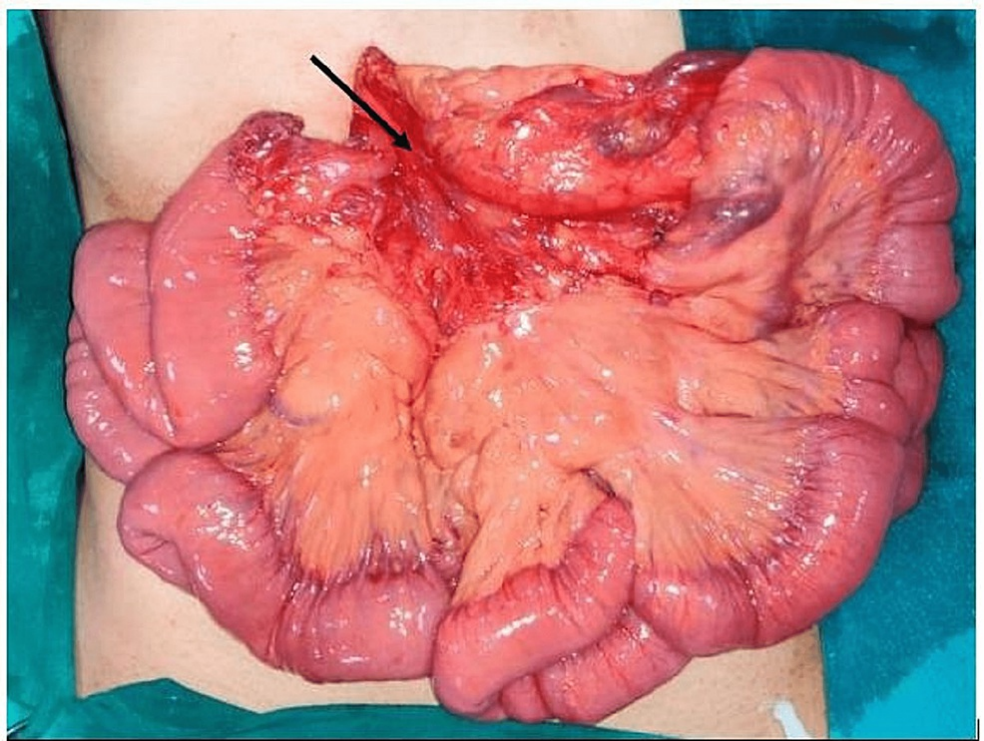
Widening of the mesentery (black arrow).

Laparoscopic derotation was attempted but converted to open exploratory laparotomy due to difficulty. Division of Ladd’s bands with derotation of midgut volvulus and widening of the mesentery was performed along with appendectomy (Figures 7-8).

The patient had an uneventful postoperative course and was discharged on postoperative day 7. He was doing well on a six-month follow-up, with improvement in symptoms and weight gain.

## Discussion

Intestinal malrotation is a condition caused due to complete or incomplete failure of the 270 degrees of counter-clockwise rotation of the midgut around the superior mesenteric axis in early fetal life. During the fourth week of fetal development, the gut forms a narrow tube with a vascular axis that divides it into foregut, midgut, and hindgut [8]. The rotation of abdominal viscera happens in three stages as follows [9]:

Stage 1: Physiological herniation of the midgut by the 4th-6th week of fetal life, followed by a 90-degree counter-clockwise rotation before returning into the abdomen by the 10th week.

Stage 2: A further rotation of 180 degrees counter-clockwise in the abdominal cavity. The related anomalies include nonrotation of the gut, malrotation, or reverse rotation.

Stage 3: Fixation of the mesentery. The related anomalies include floating cecum, duodenum, and small bowel not attached by the mesentery.

A deviation from the usual rotation of the gut results in the cecum, appendix, and large intestine to lie on the left side of the abdominal cavity, attached to the retroperitoneum on the right side by Ladd’s bands, and the location of the duodenojejunal junction on the right side due to absence of the ligament of treitz. This results in a mobile small bowel which often is prone to complications at an older age if not diagnosed in childhood, as seen in our patient who presented with midgut volvulus [10].

However, different types of rotational abnormality have been mentioned (including non-rotation, incomplete rotation, reverse rotation, and anomalous fixation of the mesentery); the most common are nonrotation or malrotation (incomplete rotation). In nonrotation, the small bowel remains on the right side and the large intestine on the left side of the peritoneal cavity with a wider mesenteric base attachment than in malrotation. So nonrotation abnormality usually goes unnoticed, whereas most patients with malrotation present with midgut volvulus or obstruction due to Ladd’s bands compression over the duodenum [9-10]. Intestinal gangrene in cases with acute midgut volvulus is a usual finding due to complete obstruction of mesenteric vessels, whereas significant collaterals form in mesenteric circulation in patients with chronic intermittent volvulus and avoid intestinal gangrene [8].

In neonates, intestinal malrotation presents acutely with bilious vomiting (93%), failure to thrive, and abdominal pain. Around 37% of infants and 12% of adults have an acute presentation in the form of intestinal volvulus and obstruction leading to bowel gangrene [11]. Owing to its rarity in adults, there are no classic symptoms or signs of malrotation. However, these patients usually present with chronic vague abdominal pain and are thus often misdiagnosed to have an acid peptic disease, irritable bowel syndrome, or psychiatric disorders [12]. Mehall et al. reported that 2% of patients with atypical symptoms like dull aching abdominal pain or reflux symptoms and 16% of patients with typical symptoms like bilious vomiting and colicky abdominal pain are said to have intestinal volvulus [13]. Thus, diagnosing patients with atypical symptoms is more challenging.

There is no gold standard investigation to diagnose malrotation in adults. Diagnostic modalities are the ultrasonogram, upper gastrointestinal contrast study, CT scan, lower GI series studies, and an MRI. The initial investigation often done is the ultrasound gram (USG) of the abdomen, which reveals an inverse relationship between SMA and SMV and also shows a classical whirlpool sign in the mid-abdomen that is diagnostic of midgut volvulus [14]. The upper gastrointestinal (GI) series is infrequently done these days, which has an accuracy of 80%. The typical findings are the vertically placed duodenum that does not cross the midline with small bowel oriented to the right side of the abdomen. Therefore, performing a combination of upper gastrointestinal study with barium enema further increases diagnostic accuracy [15]. A contrast-enhanced CT is the preferred modality when things are unclear and not correlating with symptoms with a high index of suspicion. CECT abdomen shows a corkscrew sign, whirlpool sign, and location of small bowel to the right side and large bowel to the left with absent cecum in the right iliac fossa. Demonstrating a retro mesenteric third part of the duodenum over a malpositioned duodenojejunal junction is said to be immune to developing volvulus in the future [16]. Moreover, any SMA/SMV thrombosis and development of collaterals are better visualized with a CT angiogram, as shown in our patient.

The gut's malrotation is usually treated by surgery rather than a conservative approach. Spigland et al. comment that a laparotomy is a standard treatment modality even if the patients are asymptomatic [15]. In the case of intestinal malrotation and volvulus, laparoscopic exploration is the preferred method. Compared to the traditional open Ladd's operation, laparoscopic therapy is feasible and safe without added risks [17]. The ideal principle in cases of malrotation should be the "When in doubt, open and see" approach. The gold standard surgery for this condition is a Ladd’s procedure encompassing volvulus de-rotation, Ladd’s band division, and mesentery lengthening with an appendectomy [18]. However, it is quite controversial; performing an appendectomy is the old school teaching as the division of the Ladd’s bands possesses the risk of vascularity compromise and chances of appendicitis in the future [19]. Patients with an acute presentation due to volvulus with gangrene should undergo exploratory laparotomy and resection of diseased bowel segments with anastomosis or may require stoma formation. But it is associated with a need for long-term nutritional support as short bowel syndrome is expected following this surgery.

Our case report is targeted to add to the literature as one of the few cases where the patient had SMA thrombosis with collateral development but still had no gangrene or a typical acute presentation.

## Conclusions

Malrotation of the gut in adults is a rare phenomenon. Children who are asymptomatic for malrotation and are under conservative management need frequent follow-ups as the course of the disease is unpredictable. Conservative management is thus a held responsibility of the treating surgeon. Adult patients presenting with chronic intermittent volvulus are treated by widening the base of the mesentery and division of the Ladd's bands, which reduce the risk of recurrent volvulus and relieve the compression of the malpositioned duodenojejunal junction, respectively. We advocate that a high index of suspicion is needed when surgeons deal with such cases, as timely management can lead to successful surgical outcomes for the patient.
